# Возможности применения короткой функциональной пробы с гидрохлоротиазидом в дифференциальной диагностике первичного и вторичного гиперпаратиреоза в условиях стационара

**DOI:** 10.14341/probl13150

**Published:** 2022-08-05

**Authors:** А. К. Еремкина, А. Р. Елфимова, Е. А. Абойшева, Е. В. Карасева, М. И. Фадеева, И. С. Маганева, Е. В. Ковалева, А. М. Горбачева, Е. Е. Бибик, Н. Г. Мокрышева

**Affiliations:** Национальный медицинский исследовательский центр эндокринологии; Национальный медицинский исследовательский центр эндокринологии; Национальный медицинский исследовательский центр эндокринологии; Национальный медицинский исследовательский центр эндокринологии; Национальный медицинский исследовательский центр эндокринологии; Национальный медицинский исследовательский центр эндокринологии; Национальный медицинский исследовательский центр эндокринологии; Национальный медицинский исследовательский центр эндокринологии; Национальный медицинский исследовательский центр эндокринологии; Национальный медицинский исследовательский центр эндокринологии

**Keywords:** гиперпаратиреоз, гиперкальциурия, гидрохлоротиазид, ПГПТ, ВГПТ, функциональная проба

## Abstract

**ОБОСНОВАНИЕ:**

ОБОСНОВАНИЕ. Дифференциальная диагностика между нормокальциемической формой первичного гиперпаратиреоза (нПГПТ) и вторичным гиперпаратиреозом (ВГПТ) вследствие гиперкальциурии остается актуальной клинической проблемой.

**ЦЕЛЬ:**

ЦЕЛЬ. Целью данного исследования было определение возможности использования короткой функциональной пробы с гидрохлоротиазидом для дифференциальной диагностики нПГПТ и ВГПТ.

**МАТЕРИАЛЫ И МЕТОДЫ:**

МАТЕРИАЛЫ И МЕТОДЫ. Проведено ретроспективное исследование с участием 28 пациентов c гиперпаратиреозом, нормокальциемией и гиперкальциурией, которым во время госпитализации в отделение патологии околощитовидных желез и нарушений минерального обмена ФГБУ «НМИЦ эндокринологии» Минздрава России проводили функциональную пробу с гидрохлоротиазидом 50 мг/сут. Показатели фосфорно-кальциевого обмена оценивались исходно и через 3–5 дней после инициации терапии тиазидным диуретиком.

**РЕЗУЛЬТАТЫ:**

РЕЗУЛЬТАТЫ. Согласно полученным данным, пациенты были разделены на три группы: 1-я группа (n=21) — пациенты, у которых по результатам короткой пробы с гидрохлоротиазидом диагностирован ПГПТ: спровоцирована гиперкальциемия при сохранении повышенного уровня интактного паратгормона (иПТГ) (n=19) либо нормокальциемия по уровню альбумин-скорректированного кальция (Саскорр) при нарастании уровня иПТГ (n=2). Медиана исходного уровня Саскорр. составила 2,48 ммоль/л [2,47; 2,52], медиана иПТГ — 107,5 пг/мл [86,8; 133,0], по окончании пробы Саскорр. — 2,63 ммоль/л [2,59; 2,66], иПТГ — 102,1 пг/мл [95,7; 124,1]. 2-я группа (n=1) — пациент, у которого по результатам пробы диагностирован ВГПТ: достигнуто нормальное значение иПТГ крови при сохранении нормокальциемии. Исходно: Саскорр. 2,35 ммоль/л, иПТГ 74,5 пг/мл; после пробы: Саскорр. 2,27 ммоль/л, иПТГ 50,7 пг/мл. 3-я группа (n=6) — пациенты, которым не удалось окончательно установить диагноз и было рекомендовано продолжение пробы в амбулаторных условиях. Медиана исходного Саскорр. — 2,39 ммоль/л [2,33; 2,45], медиана иПТГ — 97,0 пг/мл [83,1; 117,0]); на фоне приема гидрохлоротиазида Саскорр. — 2,47 ммоль/л [2,42; 2,48], иПТГ — 91,3 пг/мл [86,9; 124,0]. При сравнительном анализе данные группы статистически значимо отличались друг от друга только по исходным уровням Саскорр. (р=0,003, U-тест, с учетом поправки Бонферрони Р0=0,006); уровни иПТГ, суточной кальциурии, показатели фильтрационной функции почек и фосфора были сопоставимы. Также не выявлено значимых различий по частотам классических осложнений ПГПТ.

**ЗАКЛЮЧЕНИЕ:**

ЗАКЛЮЧЕНИЕ. По результатам исследования у 21 из 28 пациентов в ходе модифицированной пробы с гидрохлоротиазидом на 3–5-е сутки подтвержден ПГПТ. Полученные данные имеют высокую значимость для верификации диагноза у госпитализированных больных с неуточненным генезом гиперпаратиреоза.

## Abstract (en)

**BACKGROUND:**

BACKGROUND: Differential diagnosis between the normocalcemic primary hyperparathyroidism (nPHPT) and secondary hyperparathyroidism (SHPT) due to hypercalciuria remains a challenge.

**AIM:**

AIM: The aim of this study was to investigate the capability of short test with hydrochlorothiazide for the differential diagnosis of nPHPT and SHPT.

**MATERIALS AND METHODS:**

MATERIALS AND METHODS: A retrospective study was conducted with the participation of 28 patients who underwent a functional test with thiazide diuretics during hospitalization in the Department of parathyroid glands pathology and mineral disorders of the Endocrinology Research Centre, Russia. Parameters of mineral metabolism were evaluated before and 3–5 days after taking hydrochlorothiazide 50 mg/day.

**RESULTS:**

RESULTS: According to baseline and dynamic biochemical evaluation patients were divided into 3 groups. Group 1 (n=21) included patients with confirmed PHPT, who reached hypercalcemia accompanying with an elevated level of iPTH (n=19) or an increased level of iPTH accompanying with normocalcemia (n=2). In group 1, baseline Caadj. was 2.48 mmol/l [2.47; 2.52], iPTH 107.5 pg/ml [86.8; 133.0], after short test — 2.63 mmol/l [2.59; 2.66] and 102.1 pg/ml [95,7; 124,1]. Group 2 included only one who was diagnosed with SHPT, a normal value of iPTH with concomitant normocalcemia was achieved after 4 days of hydrochlorothiazide therapy (baseline Caadj. 2.35 mmol/l, iPTH 74.5 pg/ml vs at 2.27 mmol/l and 50.7 pg/ml respectively). Patients with doubtful results of the test entered in group 3 (n=6), they did not achieve significant changes in the calcium and iPTH levels, so it was recommended to continue the test on an outpatient basis (baseline Caadj. 2.39 mmol/l [2.33;2.45], iPTH 97.0 pg/ml [83.1;117.0]); after short test — 2.47 mmol/l [2.42; 2.48] and 91.3 pg/ml [86.9; 124.0] respectively). Groups with PHPT and SHPT and doubtful results significantly differed from each other in Caadj (р=0.003, U-test, Bonferroni correction Р0=0.006), but not in iPTH, daily calciuria, eGFR, and phosphorus. There were no significant differences in the incidence of classical complications of PHPT.

**CONCLUSION:**

CONCLUSION: The diagnosis of PHPT was confirmed in 21/28 patients 3–5 days after taking hydrochlorothiazide 50 mg/day. The obtained results are significant for the differential diagnosis in hospitalized patients with an unspecified genesis of hyperparathyroidism.

## ОБОСНОВАНИЕ

Нормокальциемический первичный гиперпаратиреоз (нПГПТ) характеризуется неизменно нормальным уровнем альбумин-скорректированного и/или ионизированного кальция (определенного прямым методом) сыворотки крови в сочетании со стойким повышением уровня интактного паратгормона (иПТГ) на основании динамических лабораторных измерений в течение более чем 3 мес при условии, что были исключены все вторичные причины гиперпаратиреоза [1][2]. Следовательно, нПГПТ представляет собой диагноз исключения и может рассматриваться только после тщательной оценки причин вторичного гиперпаратиреоза (ВГПТ).

нПГПТ считается относительно новой формой заболевания с гетерогенным фенотипом от бессимптомного течения до манифестации всех основных осложнений, при этом лежащий в основе его развития патогенетический механизм точно неизвестен. С одной стороны, он может представлять собой начальную стадию заболевания, предшествующую гиперкальциемическому варианту, с другой стороны — специфическое состояние, характеризующееся резистентностью к действию ПТГ, прежде всего со стороны почек и костей. Не исключается дополнительное влияние сопутствующего дефицита/недостаточности витамина D на поддержание стойкой нормокальциемии при нПГПТ [1].

Актуальной проблемой остается верификация диагноза нПГПТ, ведь именно от этого зависит дальнейшая лечебная тактика. Единственным методом радикального лечения нПГПТ, так же как и при гиперкальциемической форме, является паратиреоидэктомия, в то время как ВГПТ требует консервативного подхода с назначением тех или иных лекарственных препаратов. Диагностика должна быть направлена на исключение всех причин ВГПТ: дефицит/недостаточность витамина D (всоответствии с актуальными российскими клиническими рекомендациями уровень 25-OH витамина D менее 30 нг/мл) и низко-кальциевая диета, ренальная гиперкальциурия, нарушение фильтрационной функции почек (особенно при 3–5-й стадии хронической болезни почек), патология желудочно-кишечного тракта, сопровождающаяся синдромом мальабсорбции (целиакия, воспалительные заболевания кишечника, состояния после бариатрических вмешательств и др.), прием медикаментов, влияющих на секрецию ПТГ (препараты лития, блокаторы протонной помпы, бисфосфонаты, деносумаб, ингибиторы натрий-глюкозного котранспортера (Sodium-Glucose Cotransporter-2 Inhibitors, SGLT2), антиконвульсанты, диуретики). Следует также учитывать роль гипофосфатемии (в том числе в рамках фактора роста фибробластов 23 (ФРФ23)-продуцирующих опухолей) в развитии ВГПТ [1][2].

Согласно клиническим рекомендациям по ПГПТ, для дифференциальной диагностики между нПГПТ и ВГПТ пациентам с сочетанием повышенного уровня иПТГ и нормокальциемией рекомендовано проведение функциональных проб с препаратами витамина D и/или гидрохлоротиазидом. У пациентов с ПГПТ их назначение, как правило, провоцирует развитие гиперкальциемии при сохранении повышенного уровня иПТГ, а у пациентов с ВГПТ — снижение/нормализацию уровня иПТГ при нормальном уровне кальция в крови [2].

В случае наличия гиперкальциурии целесообразно проведение пробы с тиазидными диуретиками. Классический вариант данной провокационной пробы был первоначально предложен урологами для пациентов с нефролитиазом [3]. На 2 нед назначается терапия гидрохлоротиазидом в дозе 25 мг 2 раза в сутки. Показатели кальциемии и уровня иПТГ измеряются исходно и на 15-й день приема препарата. У пациентов с ренальной гиперкальциурией назначение тиазидов приводит к нормализации иПТГ в сыворотке крови при сохранении нормокальциемии. Если нормализации иПТГ не произошло, то, вероятно, имеются автономная продукция ПТГ и резорбтивная гиперкальциурия, что подтверждает диагноз ПГПТ [4].

С учетом продолжительности классическая проба с тиазидными диуретиками в основном используется в амбулаторных условиях. Однако пациенты с нормокальциемией, гиперпаратиреозом и гиперкальциурией могут быть направлены в стационар, где сроки обследования значимо меньше. Поэтому целью данной работы стала оценка модифицированной версии пробы с укорочением ее проведения до 3–5 дней у госпитализированных больных с неуточненным генезом гиперпаратиреоза.

## ЦЕЛЬ ИССЛЕДОВАНИЯ

Цель исследования — определить возможность применения короткой функциональной пробы с тиазидным диуретиком (гидрохлоротиазидом) в дифференциальной диагностике между нПГПТ и ВГПТ у госпитализированных пациентов.

## МАТЕРИАЛЫ И МЕТОДЫ

## Место и время проведения исследования

Место проведения. Исследование было проведено на базе отделения патологии околощитовидных желез и нарушений минерального обмена ФГБУ «НМИЦ эндокринологии» Минздрава России с января 2018 по июнь 2021 гг.

Время исследования. В исследование вошли пациенты, находившиеся на стационарном лечении в указанном отделении в период с 01.01.2018 по 30.06.2022.

## Изучаемые популяции

Критерии включения:

повышение сывороточной концентрации иПТГ (РИ 15–65 пг/мл) выше верхней границы референсного диапазона лаборатории;концентрация альбумин-скорректированного кальция (Саскорр) в пределах референсного диапазона лаборатории (2,15–2,55 ммоль/л);наличие гиперкальциурии (>8 ммоль/сут);скорость клубочковой фильтрации (СКФ) >60 мл/мин/1,73м2;возраст старше 18 лет.

Критерии исключения:

## Способ формирования выборки из изучаемой популяции (или нескольких выборок из нескольких изучаемых популяций)

В данной работе применялся сплошной метод формирования выборки.

## Дизайн исследования

Проведено одноцентровое интервенционное неконтролируемое ретроспективное исследование.

Во всех группах показатели фосфорно-кальциевого обмена (иПТГ, кальций общий, альбумин, фосфор, креатинин сыворотки крови, суточная кальциурия) оценивались исходно и через 3–5 дней (иПТГ, кальций общий, альбумин) после начала терапии гидрохлоротиазидом в дозе 25 мг 2 раза в сутки.

ПГПТ считался подтвержденным, если по результатам короткой пробы была достигнута гиперкальциемия (повышение Саскорр>2,55 ммоль/л) в сочетании с повышенной концентрацией иПТГ или, реже, Саскорр. оставался на верхненормальном уровне, нов сочетании с нарастанием уровня иПТГ. ВГПТ считался подтвержденным, если по результатам короткой пробы была достигнута нормализация концентрации иПТГ (<65 пг/мл) при сохраняющейся нормокальциемии. В 3-ю группу вошли пациенты, укоторых через 3–5 сут сохранялась нормокальциемия (концентрация Саскорр в диапазоне 2,15–2,55 ммоль/л) при снижении, но не нормализации уровня иПТГ, что не позволяло однозначно установить диагноз.

Наличие осложнений ПГПТ устанавливалось в соответствии с утвержденными Федеральными клиническими рекомендациями на основании выполненных исследований (ультразвуковое исследование (УЗИ) почек, двухэнергетическая рентгеновская денситометрия (DXA) поясничного отдела позвоночника (LI–LIV), проксимального отдела бедренной (total hip, neck) и лучевой костей (radius total, radius 33%), рентгенография грудного и поясничного отделов позвоночника в боковой проекции при наличии показаний к исследованию). За клинически значимое поражение костной ткани было принято снижение минеральной плотности кости ниже –2,0 SD по Z-критерию для женщин репродуктивного возраста и мужчин моложе 50 лет и менее –2,5 SD по Т-критерию для женщин в постменопаузе и мужчин старше 50 лет в любом из указанных отделов. Поражение почек устанавливалось при наличии признаков нефрокальциноза/нефролитиаза по данным УЗИ почек.

## Описание медицинского вмешательства (для интервенционных исследований)

Всем пациентам после выполнения первичного лабораторного обследования назначался гидрохлоротиазид в дозе 25 мг 2 раза в сутки в течение 3–5 дней, далее проводились повторная оценка показателей фосфорно-кальциевого обмена и принятие решения о прекращении или продолжении пробы с тиазидным диуретиком.

## Методы

Биохимические показатели сыворотки крови (кальций общий (референсный интервал (РИ) 2,15–2,55 ммоль/л), альбумин (РИ 34–48 г/л для женщин, 35–50 г/л для мужчин), фосфор (РИ 0,74–1,52 ммоль/л), креатинин (РИ 50–98 мкмоль/л для женщин, 63–110 мкмоль/л для мужчин) исследованы на автоматическом биохимическом анализаторе ARCHITECH с8000 (Abbott, CША). Пересчет концентрации кальция крови с поправкой на уровень альбумина проводился по формуле:

Саскорр, ммоль/л = измеренный уровень кальция сыворотки (ммоль/л)+0,02×(40 − измеренный уровень альбумина, г/л).

Расчетная скорость клубочковой фильтрации (рСКФ) определялась с учетом возраста и уровня креатинина сыворотки по формуле CKD-EPI 2009. Определение иПТГ крови (РИ 15–65 пг/мл) проводилось на электрохемилюминесцентном анализаторе Cobas 6000 (Roche, Германия). Уровень кальция в суточной порции мочи (РИ 2,5–8,0 ммоль/л) исследовался на автоматическом биохимическом анализаторе ARCHITECH с8000. DXA проводилась на денситометре Lunar iDXA (GE Healthcare, США). УЗИ почек проводилось на аппаратах Voluson Е8 датчиками RAB 6-D, С1–5 (GE Healthcare, США) или Aplio 500 датчиком 6С1 (Toshiba, Япония). Рентгенография грудного и поясничного отделов позвоночника в прямой и боковой проекциях проводилась на аппарате Axiom Iconos R 200 (Siemens, Германия).

## Статистический анализ

Статистический анализ был выполнен с использованием программы Statistica v. 13.3 (TIBCO Software Inc., США). При сравнении двух независимых групп между собой по количественным признакам был применен критерий Манна–Уитни (U-тест), покачественным показателям — точный критерий Фишера. При сравнении двух зависимых групп по количественным признакам был применен критерий Вилкоксона. Критический уровень статистической значимости при проверке статистических гипотез принят равным 0,05. При множественных сравнениях применялась поправка Бонферрони путем коррекции критического уровня значимости. Количественные данные представлены в виде медианы и интерквартильных интервалов Me [ Q1; Q3], качественные — в виде абсолютных и относительных частот n (%).

## Этическая экспертиза

Проведение исследования было одобрено комитетом по этике ФГБУ «НМИЦ эндокринологии» Минздрава России (протокол № 1 от 25.01.2017).

## РЕЗУЛЬТАТЫ

В соответствии с критериями включения и исключения в исследование включены 28 пациентов: 1 мужчина (3,6%) и 27 женщин (96,4%). Медиана возраста пациентов в общей группе составила 59 [ 53; 66] лет.

Согласно полученным показателям фосфорно-кальциевого обмена, пациенты разделены на три группы: 1-я группа (n=21) — пациенты, у которых по результатам короткой пробы с гидрохлоротиазидом диагностирован ПГПТ; 2-я группа (n=1) — пациент, у которого по результатам короткой пробы диагностирован ВГПТ; 3-я группа (n=6) — пациенты, которым по результатам короткой пробы установить диагноз однозначно не удалось. В последующем для проведения сравнительного анализа группы 2 и 3 были объединены (рис. 1) в единую группу (с учетом их малочисленности).

**Figure:**
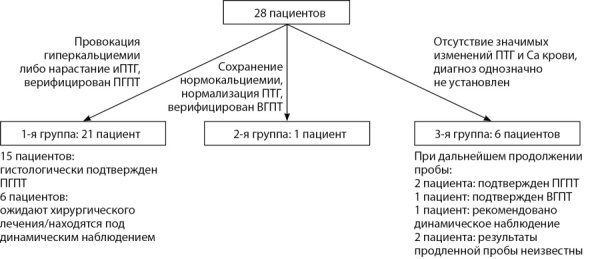
Рисунок 1. Разделение пациентов по группам по результатам исследования.Figure 1. The division of patients into groups according to the results of the study.

В 1-й группе по результатам функциональной пробы с тиазидным диуретиком у большинства пациентов была спровоцирована гиперкальциемия (повышение Саскорр.>2,55 ммоль/л) при сохранении повышенного уровня иПТГ (n=19), у 2 пациентов сохранялась нормокальциемия по уровню Саскорр. при нарастании уровня иПТГ, что было расценено как ПГПТ. Медиана исходного уровня Саскорр. составила 2,48 ммоль/л [ 2,47; 2,52], медиана иПТГ — 107,5 пг/мл [ 86,8; 133,0]. Другие лабораторные показатели фосфорно-кальциевого обмена представлены в таблице 1. Структурные изменения в почках по типу нефролитиаза/нефрокальциноза определялись в 52% случаев, снижение минеральной плотности костей до уровня остеопении иостеопороза — в 4,8 и 61,9%, при этом низкоэнергетические переломы отмечены у 19% пациентов. На фоне пробы с гидрохлоротиазидом через 3–5 дней отмечалось статистически значимое повышение уровня кальция (при сравнении Саскорр. до пробы ичерез 3–5 дней, p<0,001, критерий Вилкоксона, с учетом поправки Бонферрони Р0=0,025) при сохранении повышенного уровня иПТГ (при сравнении иПТГ до пробы и через 3–5 дней после; p=0,958). Медиана Саскорр. составила 2,63 ммоль/л [ 2,59; 2,66], медиана иПТГ — 102,1 пг/мл [ 95,7; 124,1]. 15 пациентов из данной группы впоследствии были прооперированы, и диагноз ПГПТ был верифицирован гистологическим исследованием (у 14 больных — аденома околощитовидных желез (ОЩЖ), у 1 пациентки — множественные гиперплазии) в сочетании с нормализацией иПТГ в послеоперационном периоде. Три пациента из данной группы ожидают хирургического лечения, оставшиеся в связи с бессимптомным течением ПГПТ находятся под динамическим наблюдением.

**Table:** Таблица 1. Характеристика основных показателей фосфорно-кальциевого обмена до и через 3–5 дней после приема гидрохлоротиазидаTable 1. Characteristics of the main indicators of phosphorus-calcium metabolism before and 3–5 days after taking hydrochlorothiazide

Группы	N	До пробы	На пробе (через 3–5 дней)
иПТГ, пг/мл	Саскорр., ммоль/л	Р, ммоль/л	Са суточной мочи, ммоль/сут	СКФ (CKD-EPI), мл/мин/1,73м2	25(ОН)D, нг/мл	иПТГ, пг/мл	Саскорр., ммоль/л
1	21	107,5 [ 86,8; 133,0]	2,48 [ 2,47;2, 52]	0,93 [ 0,87; 0,97]	9,4 [ 8,7; 11,9]	82 [ 77; 87]	30,8 [ 20,8; 43,5]	102,1 [ 95,7; 124,1]	2,63 [ 2,59; 2,66]
2	1	74,5	2,35	0,9	13,05	111	33,25	50,7	2,27
3	6	97 [ 83,1; 117,0]	2,39 [ 2,33; 2,45]	1,03 [ 1; 1,08]	10,5 [ 9,3; 11,6]	85,8 [ 82,9; 86]	33 [ 31,5; 48]	91,3 [ 86,9; 124]	2,47 [ 2,42; 2,48]

Подтвердить диагноз ВГПТ на фоне короткой пробы с гидрохлоротиазидом удалось только у 1 пациента, у которого отмечалась нормализация концентрации иПТГ на фоне стойкой нормокальциемии по уровню Саскорр. (исходно Саскорр. 2,35 ммоль/л, иПТГ 74,5 пг/мл; на 4-е сутки после инициации терапии гидрохлоротиазидом — Саскорр. 2,27 ммоль/л, иПТГ 50,7 пг/мл). До начала пробы уровень витамина D составил 33,25 нг/мл. Из сопутствующих заболеваний можно отметить наличие микронефролитиаза, а также снижение минеральной плотности костной ткани относительно ожидаемых возрастных значений. Пациент продолжает амбулаторное наблюдение, на фоне поддерживающей дозы гидрохлоротиазида 25 мг достигнуты нормокальциемия, нормокальциурия и стойкое поддержание иПТГ в референсном диапазоне.

В 3-й группе результаты короткой пробы не позволяли однозначно установить диагноз, что в большинстве случаев потребовало продолжения пробы с гидрохлоротиазидом в суточной дозе 50 мг. Медиана Саскорр. исходно составила 2,39 ммоль/л [ 2,33;2,45], медиана иПТГ 97,0 пг/мл [ 83,1; 117,0]); на 3–5 сутки после начала пробы — медиана Саскорр. составила 2,47 ммоль/л [ 2,42; 2,48], медиана иПТГ — 91,3 пг/мл [ 86,9; 124,0]). Статистически значимых различий по уровню иПТГ и Саскорр. получено не было (p=0,753 и р=0,5 соответственно). Другие лабораторные показатели фосфорно-кальциевого обмена представлены в таблице 1. Структурные изменения в почках по типу нефролитиаза/нефрокальциноза наблюдались в 66,7% случаев, снижение минеральной плотности костей до уровня остеопении/остеопороза выявлены у большинства пациентов (33,3/50%), при этом низкоэнергетические переломы отмечены у 1 пациента. Всем пациентам было рекомендовано продолжить пробу сгидрохлоротиазидом 50 мг/сут до 2 нед, однако сведения имеются только о 4 из них. У двух был подтвержден ПГПТ, пациентки были прооперированы, по результатам гистологического исследования верифицирована аденома ОЩЖ. У 1 больной через 2 нед была достигнута нормализация иПТГ с сохранением нормокальциемии, что позволило установить диагноз ВГПТ. Одной пациентке рекомендован динамический контроль, так как результаты пробы были интерпретированы как сомнительные — значимое снижение иПТГ относительно исходных значений (но не нормализация) с сохранением средненормальных показателей Саскорр. У остальных пациентов (n=2) исход неизвестен в связи с тем, что они продолжили наблюдение амбулаторно поместу жительства и не предоставили результаты лабораторных анализов в динамике.

Далее нами был проведен сравнительный анализ между пациентами 1-й и объединенной группы (2+3). При сравнении группы статистически значимо отличались друг от друга только по исходным уровням Саскорр. (2,48 ммоль/л [ 2,47; 2,52] против 2,35 ммоль/л [ 2,32; 2,45] соответственно; р=0,003, U-тест, с учетом поправки Бонферрони Р0=0,006), при этом различий в концентрациях иПТГ, суточной кальциурии, рСКФ, фосфора не выявлено. Кроме того, группы были сопоставлены по частотам классических осложнений ПГПТ.

## ОБСУЖДЕНИЕ

ВГПТ и нПГПТ имеют аналогичную биохимическую картину, хотя патогенез данных заболеваний и, как следствие, подходы к лечению значимо различаются. Нормокальциемический вариант ПГПТ развивается в результате автономной гиперсекреции ПТГ одной или несколькими ОЩЖ. При вторичном генезе гиперпаратиреоза повышение уровня гормона является реакцией на снижение сывороточного кальция вследствие различных состояний. Секреция ПТГ остается повышенной до тех пор, пока неустранены причины, связанные с недостаточным поступлением и/или усиленным выведением солей кальция из организма, на что и должна быть направлена терапия ВГПТ. Лечение нПГПТ заключается в нормализации секреции иПТГ путем паратиреоидэктомии.

Гиперкальциурия — одна из основных причин избыточного выведения кальция из организма. Большая часть отфильтрованного кальция реабсорбируется в нефроне. Этот процесс включает два основных этапа: 1) кальций пассивно реабсорбируетсяв проксимальных канальцах и петле Генле по электрохимическому градиенту, создаваемому реабсорбцией натрия и воды; 2) активный транспорт кальция в соответствии с изменениями его баланса в дистальном канальце и прилегающем соединительном сегменте (участок между дистальным канальцем и кортикальным собирательным канальцем). ПТГ и кальцитриол стимулируют этот активный процесс. На реабсорбцию ионов кальция и их экскрецию с мочой может влиять назначение диуретиков. Экскреция кальция увеличивается при приеме петлевых диуретиков и снижается при приеме тиазидных препаратов и амилорида. То, как проявляются эти эффекты, связано с механизмами транспорта натрия, хлоридов и кальция вразличных сегментах, чувствительных к данным лекарственным средствам [5][6].

ВГПТ, вызванный гиперкальциурией, может быть результатом избыточного потребления продуктов, содержащих соли натрия, чая и кофе, приема фуросемида (наиболее часто используемого петлевого диуретика), поэтому тщательный сбор анамнеза позволит исключить эти причины. Существуют генетические дефекты, вызывающие гиперкальциурию (мутации в генах CLDN16, CLCN5, TRPV5, OCRL1, SLC34A3/NPT2c, SCL34A1/NPT2a, SLC9A3R1/NHERF1, NKCC2, ROMK1), однако они встречаются редко, и выявляются, как правило в детском и подростковом возрасте, в ходе генетического тестирования [7].

Провокационный тест с тиазидными диуретиками помогает дифференцировать нПГПТ от ренальной гиперкальциурии. Гидрохлоротиазид и другие препараты данной группы ингибируют транспортный белок, обеспечивающий перенос Na+ и Сl– в клетки канальцевого эпителия, вследствие чего снижается реабсорбция этих ионов в дистальных отделах канальцев. Эти препараты усиливают выведение с мочой калия, магния, гидрокарбонатов и фосфатов, при этом задерживают в организме ионы кальция и ураты. Несмотря на то, что классический вариант 2-недельной провокации с тиазидами (“thiazide challenge”) для дифференциальной диагностики гиперпаратиреоза был описан в 2009 г. B.H. Eisner и соавт., попытки использовать тиазиды с этой целью предпринимались и раньше [8][9]. Так, в работе 1977 г. описано использование гидрохлоротиазида в дозе 50 мг каждые 8 ч в течение 4 дней у пациентов с пограничными уровнями кальциемии, при этом проводилась динамическая оценка уровней иПТГ и кальция, на основании которой устанавливался окончательный диагноз и принималось решение о дальнейшей тактике [9].

Проба с тиазидными диуретиками имеет ряд существенных ограничений. Не рекомендуется ее проведение у пациентов со снижением рСКФ менее 60 мл/мин/1,73 м2. С одной стороны, хроническая болезнь почек 3–5-й стадии сама по себе является причиной повышения уровня иПТГ, что затрудняет интерпретацию полученных результатов. С другой — клиренс креатинина менее 30 мл/мин является абсолютным противопоказанием к приему препарата [3].

В большинстве случаев в результате модифицированной короткой пробы с гидрохлоротиазидом нами был подтвержден первичный генез гиперпаратиреоза (у 21 из 28 пациентов, 75%). Данная провокация, как правило, приводит к развитию гиперкальциемии (в том числе на 3–5-е сутки после инициации пробы), что является ценным диагностическим критерием, помимо отсутствия нормализации иПТГ. Несмотря на отсутствие гиперкальциемии на 3–5-е сутки, повышение уровня иПТГ отисходных значений также может быть расценено как проявление ПГПТ. При сравнении лиц с нПГПТ и объединенной группы (ВГПТ + сомнительные результаты пробы) были выявлены статистически значимые различия только по исходным уровням Саскорр, при этом различий по другим параметрам фосфорно-кальциевого обмена и рСКФ выявлено не было. Как в случае нПГПТ, так и при ВГПТ фиксировались структурные изменения в почках, костная патология, что не позволяет использовать наличие классических осложнений ПГПТ в дифференциальной диагностике. Наши результаты в целом согласуются с данными ретроспективного анализа М. Griebeler и соавт., посвященному случаям гиперкальциемии, ассоциированной с приемом тиазидных диуретиков [10]. Частота выявления гиперкальциемии в исследовании коррелировала с частотой ПГПТ в городе, где проводился набор пациентов для исследования. Среди всех пациентов (221) с тиазид-ассоциированной гиперкальциемиейу 24% пациентов был позднее диагностирован ПГПТ. Основываясь на сохранении гиперкальциемии после окончания приема тиазидов, авторы предположили, что всего в исследуемой когорте ПГПТ мог быть причиной гиперкальциемии в 71% случаев [10].

В случае если по результатам пробы у пациента верифицирован диагноз ВГПТ, дальнейшее наблюдение рекомендуется у уролога и/или нефролога. Для коррекции гиперкальциурии может быть рассмотрен вопрос о возможности терапии тиазидными диуретиками в долгосрочной перспективе. Эффективность данной терапии в отношении снижения суточной кальциурии и, следовательно, прогрессирования нефролитиаза и рецидива почечных колик была неоднократно продемонстрирована в ряде рандомизированных клинических исследований [4].

## Клиническая значимость результатов

Впервые проведено изучение возможности применения короткой функциональной пробы с гидрохлоротиазидом в дифференциальной диагностике между нПГПТ и ВГПТ вследствие синдрома гиперкальциурии в условиях стационара. Полученные результаты имеют высокую значимость для верификации диагноза у госпитализированных больных с неуточненным генезом гиперпаратиреоза.

## Ограничения исследования

Сохранение нормокальциемии при повышенном уровне иПТГ у пациентов из 3-й группы не позволяло однозначно установить диагноз за короткий период наблюдения. У ряда пациентов из представленной выборки не было достигнуто целевого уровня 25(ОН) витамина D>30 нг/мл.

## Направления дальнейших исследований

Увеличение мощности исследования.

## ЗАКЛЮЧЕНИЕ

Впервые проведено изучение возможности применения короткой провокационной пробы с гидрохлоротиазидом в дифференциальной диагностике между нПГПТ и ВГПТ вследствие гиперкальциурии в условиях стационара. Полученные результаты имеют высокую значимость для верификации диагноза у госпитализированных больных с неуточненным генезом гиперпаратиреоза.

## ДОПОЛНИТЕЛЬНАЯ ИНФОРМАЦИЯ

Источники финансирования. Данное исследование выполнено в рамках государственного задания «Оптимизация Российского электронного реестра пациентов с первичным гиперпаратиреозом», регистрационный номер 121030100032-7.

Конфликт интересов. Авторы декларируют отсутствие явных или потенциальных конфликтов интересов.

Участие авторов. Еремкина А.К., Мокрышева Н.Г. — концепция и дизайн исследования; Еремкина А.К. — сбор и обработка материала; Елфимова А.Р. — статистический анализ данных; Еремкина А.К., Карасева Е.В., Абойшева Е.А. — анализ литературных данных; Еремкина А.К., Карасева Е.В., Абойшева Е.А., Елфимова А.Р., Горбачева А.М., Бибик Е.Е., Ковалева Е.В., Фадеева М.И., Маганева И.С. — написание основного текста и редактирование статьи; Мокрышева Н.Г. — внесение правок и финальное редактирование. Все авторы одобрили финальную версию статьи перед публикацией, выразили согласие нести ответственность за все аспекты работы, подразумевающую надлежащее изучение и решение вопросов, связанных с точностью или добросовестностью любой части работы.

## References

[cit1] Bollerslev Jens, Rejnmark Lars, Zahn Alexandra, Heck Ansgar, Appelman-Dijkstra Natasha M, Cardoso Luis, Hannan Fadil M, Cetani Filomena, Sikjaer Tanja, Formenti Anna Maria, Björnsdottir Sigridur, Schalin-Jäntti Camilla, Belaya Zhanna, Gibb Fraser, Lapauw Bruno, Amrein Karin, Wicke Corinna, Grasemann Corinna, Krebs Michael, Ryhänen Eeva, Makay Özer, Minisola Salvatore, Gaujoux Sébastien, Bertocchio Jean-Philippe, Hassan-Smith Zaki, Linglart Agnès, Winter Elizabeth M, Kollmann Martina, Zmierczak Hans-Georg, Tsourdi Elena, Pilz Stefan, Siggelkow Heide, Gittoes Neil, Marcocci Claudio, Kamenický Peter, _ _, Carola Zillikens, Morten Frost, Lars Rolighed, Antonio Sitges-Serra, Sabrina Corbetta, Brigitte Decallonne, Iuliana Gherlan, Laura Gianotti, Daniel Grigorie, Elif Hindié, Mairead Kiely, Kirsten Lindner, Polyzois Makras, Barbara Obermayer-Pietsch, Fastino R Perez-Lopez, Mikkel Pretorius, Federica Saponaro, Christian Trummer, Kyriakos Vamvakidis, Natia Vashakmadze, Maria P Yavropoulou (2021). European expert consensus on practical management of specific aspects of parathyroid disorders in adults and in pregnancy: recommendations of the ESE Educational Program of Parathyroid Disorders (PARAT 2021). European Journal of Endocrinology.

[cit2] Mokrysheva NG, Eremkina AK, Mirnaya SS, et al. The clinical practice guidelines for primary hyperparathyroidism, short version. Problems of Endocrinology. 2021;67(4):94-124. doi: https://doi.org/10.14341/probl12801 34533017 10.14341/probl12801 PMC9753843

[cit3] Eisner Brian H., Ahn Jennifer, Stoller Marshall L. (2009). Differentiating Primary from Secondary Hyperparathyroidism in Stone Patients: The “Thiazide Challenge”. Journal of Endourology.

[cit4] Mirnaya S. S., Eremkina A. K. (2022). Hypercalciuria and hyperparathyroidism — is there always a connection?. Obesity and metabolism.

[cit5] Edwards Aurélie, Bonny Olivier (2018). A model of calcium transport and regulation in the proximal tubule. American Journal of Physiology-Renal Physiology.

[cit6] Alexander R. Todd, Dimke Henrik (2017). Effect of diuretics on renal tubular transport of calcium and magnesium. American Journal of Physiology-Renal Physiology.

[cit7] Corbetta Sabrina (2018). Normocalcemic Hyperparathyroidism. Parathyroid Disorders.

[cit8] Coe FL, Canterbury JM, Firpo JJ, Reiss E. Evidence for Secondary Hyperparathyroidism in Idiopathic Hypercalciuria. J Clin Invest. 10.1172/JCI107156 PMC302235 4682379

[cit9] COFFEY ROBERT J., LEE THOMAS C., CANARY JOHN J. (2006). The Surgical Treatment of Primary Hyperparathyroidism. Annals of Surgery.

[cit10] Griebeler Marcio L., Kearns Ann E., Ryu Euijung, Thapa Prabin, Hathcock Matthew A., Melton L. Joseph, Wermers Robert A. (2016). Thiazide-Associated Hypercalcemia: Incidence and Association With Primary Hyperparathyroidism Over Two Decades. The Journal of Clinical Endocrinology & Metabolism.

